# Paranasal Sinus Fractures in Pediatric Head Trauma: A Retrospective CT-Based Analysis of Pattern Distribution, Mechanism Correlation and Associated Intracranial Injury

**DOI:** 10.3390/diagnostics16213638

**Published:** 2026-11-04

**Authors:** Cristina-Mihaela Popescu, Ada Ștefănescu, Irina Profir, Dorin Ioan Cocoș, Roxana-Cristina Mehedinți, Cristian Constantin Budacu

**Affiliations:** 1 Medical and Pharmaceutical Research Center, Faculty of Medicine and Pharmacy, “Dunărea de Jos” University of Galați, 800008 Galati, Romania; cristina.popescu@ugal.ro (C.-M.P.); ada.stefanescu@ugal.ro (A.Ș.); cdorin1123@gmail.com (D.I.C.); roxana.mehedinti@ugal.ro (R.-C.M.); 2 Department of Morphology and Functional Sciences, Faculty of Medicine and Pharmacy, “Dunărea de Jos” University of Galați, 800008 Galați, Romania; 3 “Sf. Ioan” Clinical Emergency Pediatric Hospital, 800487 Galati, Romania; 4 Dental-Medicine Department, Faculty of Medicine and Pharmacy, “Dunărea de Jos” University of Galați, 800201 Galati, Romania; 5 Clinical Medical Department, Faculty of Medicine and Pharmacy, “Dunărea de Jos” University of Galați, 800216 Galati, Romania; 6 Faculty of Dental Medicine, “Grigore T. Popa” University of Medicine and Pharmacy from Iasi, 700115 Iasi, Romania; cristian.budacu@umfiasi.ro

**Keywords:** child, facial injuries, frontal sinus, maxillary sinus, paranasal sinuses, skull fractures, tomography

## Abstract

**Background/Objectives**: Pediatric paranasal sinus fractures are often reported within broader craniofacial trauma series. This retrospective cross-sectional descriptive study focused on fracture distribution across the four sinus groups, injury mechanisms, initial management, and associated traumatic intracranial hemorrhage. **Methods**: Written head CT reports and clinical records from a pediatric emergency hospital in Romania were reviewed retrospectively for 2021–2025. Thirty-eight patients with documented sinus fractures were included. Sinus involvement was coded by anatomical group, with bilateral involvement counted once per group. Traumatic intracranial hemorrhage was defined as hemorrhage documented on the index CT. Analyses were descriptive. **Results**: Median age was 13 years; 25 patients (65.8%) were male. Maxillary fractures occurred in 31/38 patients (81.6%), frontal fractures in 13 (34.2%), ethmoid fractures in 12 (31.6%), and sphenoid fractures in four patients (10.5%). Eighteen patients had isolated maxillary fractures, three had isolated frontal fractures, and 17 (44.7%) had multisinus fractures. Motor vehicle accidents accounted for 19 injuries (50.0%). Traumatic intracranial hemorrhage was documented in 10 patients (26.3%), including two of seven with posterior table involvement. Eight hemorrhage cases lacked posterior table fractures. Twenty patients received conservative management, 10 underwent other operative interventions, and eight were transferred; no sinus-specific operation was documented at the study institution. Among the 30 non-transferred patients, median hospital stay was six days. **Conclusions**: Maxillary involvement predominated in this selected pediatric sinus-fracture cohort. Traumatic intracranial hemorrhage occurred with both isolated and multisinus fractures and was usually documented without posterior table involvement. Report-based ascertainment, exclusions with unknown fracture status, and unavailable post-transfer outcomes limit interpretation. These findings support further descriptive investigation in larger cohorts with standardized image review.

## 1. Introduction

Traumatic brain injury remains a major cause of death and disability worldwide, with regional differences in injury burden and mechanism [1]. In children, neurological injury and craniofacial trauma require coordinated assessment because skeletal injury may coexist with brain injury and other systemic traumas [2,3,4]. Paranasal sinus fractures occupy the anatomical interface between the facial skeleton and cranial base, making their distribution relevant to both radiological documentation and acute management.

Pediatric facial fracture studies describe age-dependent patterns, associated injuries, and resource use, but their anatomical categories usually concern facial bones or craniofacial regions rather than individual sinus walls [5,6,7]. Longitudinal work also shows that fracture patterns and mechanisms change over time and across settings [8]. A sinus-by-sinus description can therefore complement broader craniofacial classifications without assuming that bone-based and sinus-based frequencies are interchangeable.

The developing paranasal sinuses differ from their adult counterparts in size, pneumatization, and relationships to the growing craniofacial skeleton. Normal developmental appearances must be recognized on CT (computed tomography) and MRI (magnetic resonance imaging) to avoid misinterpretation [9,10]. MRI-based measurements and CT-based frontal sinus morphometry demonstrate age-related growth alongside substantial interindividual variation [11,12]. Surgical anatomical studies of the pediatric sellar and parasellar regions further describe maturation of the sphenoid sinus and adjacent skull base structures [13]. Age alone cannot establish whether a sinus cavity is present or absent in an individual child; an identifiable sinus wall must be distinguished from the surrounding frontal or sphenoid bone.

The sinus walls are closely related to the orbit, anterior cranial fossa, and skull base. Frontal fracture assessment therefore considers the anterior table, posterior table, and outflow tract separately, together with displacement, cerebrospinal fluid leakage, and associated injuries [14,15]. Clinical studies of frontal sinus trauma address neurological injury and multidisciplinary management, although adult findings require cautious application to children [16,17]. A frontal sinus fracture, clinical TBI (traumatic brain injury), and CT-documented traumatic intracranial hemorrhage are distinct endpoints.

A dedicated pediatric frontal sinus series describes concomitant injuries and management algorithms [18,19]. Other studies distinguish frontal bone fractures from injuries extending to the sinus or orbital roof, and historical pediatric series document a substantial associated injury burden [20,21]. Pediatric orbital cohorts provide additional developmental context, particularly for the changing distribution of roof and floor injuries [22]. These studies offer important comparisons but do not use the same sinus-group denominator as a cohort encompassing maxillary, frontal, ethmoid, and sphenoid fractures.

CT provides acute assessment of osseous injury and intracranial hemorrhage [23]. Pediatric research comparing head and facial CT demonstrates that examination coverage and technique affect the facial injuries identified [24]. MRI may characterize additional intracranial lesions, but it was not systematically available in the present dataset [25]. Accordingly, a study based on head CT reports must distinguish documented findings from injuries established through independent review of the complete imaging dataset.

The present study aimed to describe the distribution, laterality, and extent of documented paranasal sinus fractures in a five-year pediatric head trauma cohort. Secondary objectives were to describe mechanisms, overlapping morphological features, associated hemosinus and orbital findings, traumatic intracranial hemorrhage, initial management, and hospital stay among patients treated locally. Comparisons were descriptive and were not designed to establish causal or predictive relationships. The anatomical grouping used for analysis is an operational definition, rather than a proposed or validated pediatric fracture classification.

## 2. Materials and Methods

### 2.1. Ethical Considerations. Study Design and Setting

This retrospective study was conducted at “Sf. Ioan” Clinical Emergency Pediatric Hospital, Galați, Romania, and covered January 2021 through December 2025. The institutional Ethics Committee approved the study (protocol 6824/8 May 2026) and waived informed consent for retrospective use of de-identified clinical data. Reporting was structured with reference to STROBE (STrengthening the Reporting of OBservational studies in Epidemiology) [26]; the completed checklist is provided as Supplementary Table S1.

### 2.2. Case Identification and Eligibility

Head CT reports were reviewed to identify patients aged 18 years or younger with a documented fracture involving at least one paranasal sinus wall. Eligible examinations were performed at the study institution in a trauma setting. Only the first CT examination within the same hospitalization was included. Non-trauma examinations, repeated examinations within the same hospitalization, reports without a sinus fracture, and records lacking the information required for eligibility assessment were excluded. No Le Fort pattern was reported; no separate Le Fort exclusion was applied.

The screening record contained 2603 head CT examinations, including 1428 obtained in a trauma setting. Forty-seven repeated examinations were excluded. Sixty-five records were excluded as incomplete: the mechanism of injury or the admission GCS score was missing, or the CT report described intracranial findings only and did not mention fractures at any location. These records were classified as having an unknown fracture status. Among the remaining 1316 records, 1278 had no reported paranasal sinus fracture, including reports that described a fracture only as possible or suspected, and 38 formed the cohort. Screening numbers are examination-level counts; the included cohort consists of patient records. The resulting fraction of included cases is not a population incidence estimate.

### 2.3. Data Sources and Clinical Variables

**Figure 1:**
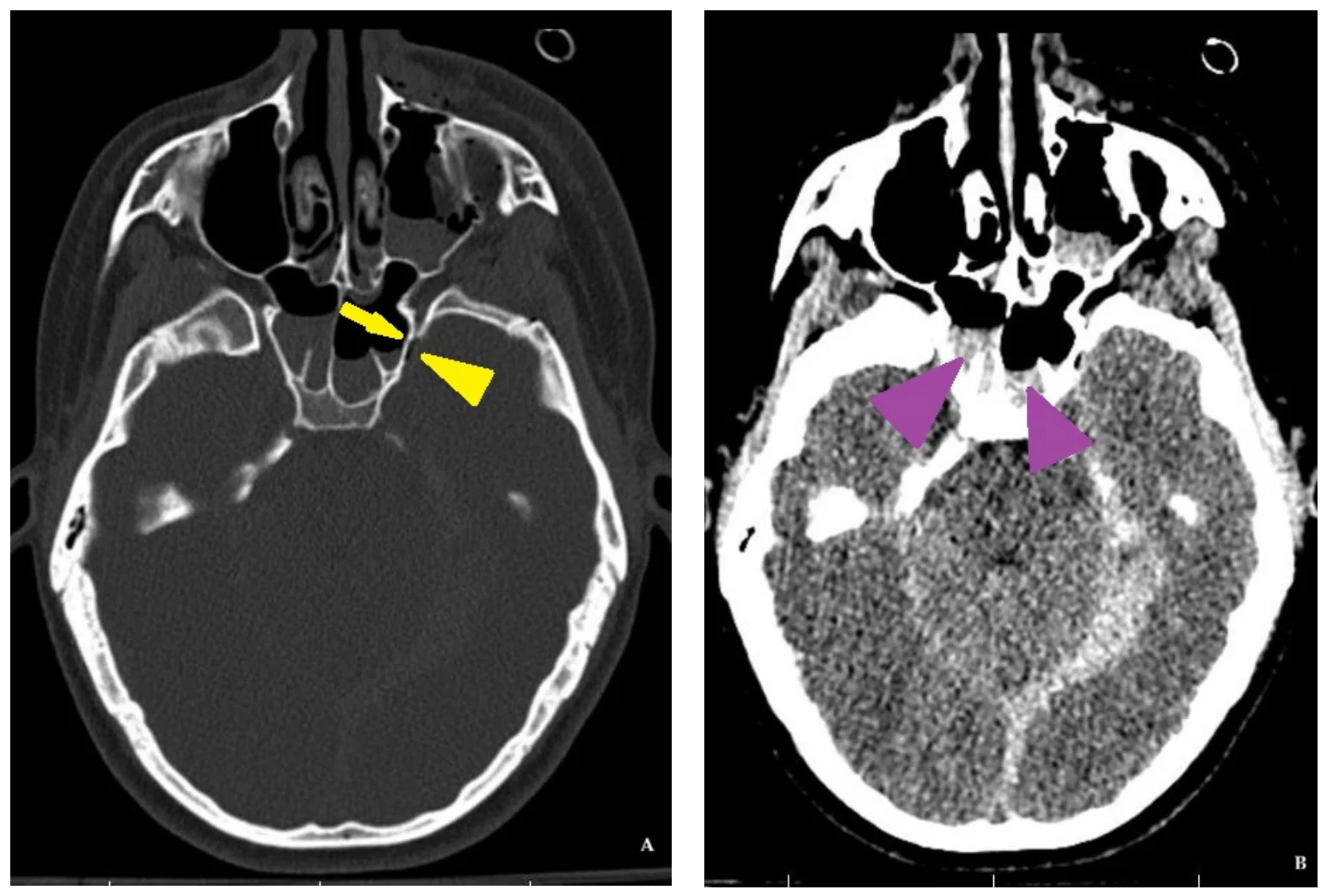
Head CT of a patient under 15 years old brought to the emergency department after an MVA (passenger). (**A**) Axial bone windowing showing left sphenoid sinus fracture (yellow arrow) with associated pneumocephalus (yellow arrowhead); no fracture in the right sphenoid sinus. (**B**) Axial brain windowing showing bilateral sphenoid hemosinus (purple arrowheads). The case is used only for illustration and was not used for data extraction.

Imaging data were abstracted from written CT reports by one reviewer with pediatric radiology expertise. An independent retrospective PACS (Picture Archiving and Communication System) image review was not performed because institutional infrastructure changes, including server migration in 2023, prevented access to the complete imaging dataset. Examinations performed after the server migration remained retrievable, and one is shown in Figure 1 for illustration only. Each report was coded against the definitions in Section 2.4. Findings described as possible or suspected were coded as not reported, and unclear laterality was checked against the clinical information on the CT request form. Extraction was performed once, by one reader, without a measure of agreement, and was not an independent blinded image interpretation.

Clinical variables were year, season, age, sex, urban or rural residence, injury mechanism, admission Glasgow Coma Scale (GCS) score, initial management, and length of hospital stay (LOS). Mechanisms were categorized as motor vehicle accident involving a passenger or pedestrian, micromobility injury, fall from height, fall from standing height, sports injury, animal-related injury, or assault. Micromobility combined bicycle and scooter injuries. Residence describes the patients included in this cohort; catchment population and exposure denominators were unavailable.

### 2.4. Imaging Definitions

Fractures were coded separately for the maxillary, frontal, ethmoid, and sphenoid sinus groups, with right, left, and bilateral involvement recorded. Frontal posterior table involvement was a separate variable. Bilateral involvement was counted once when calculating the number of fractured sinus groups. “Isolated” indicates fracture of only one sinus group and does not exclude associated orbital, facial, cranial, intracranial, or extracranial injuries. Frontal bone fractures alone were not treated as sinus fractures; frontal sinus coding required documented wall involvement, including hypoplastic sinuses.

Linear, comminuted, and depressed features were recorded as overlapping patient-level findings rather than mutually exclusive dominant patterns. These variables describe reported sinus and contiguous facial skeletal fracture morphology; they are not fracture-level counts or a validated pediatric classification. Morphology attributable solely to separate neurocranial fractures was excluded from these variables. The same overlapping definitions were used in the overall and subgroup tables.

Hemosinus was coded by sinus group and side. Orbital content herniation referred to documented orbital fat or soft-tissue herniation into an adjacent sinus, rather than displacement of a bone fragment. Nasal bone fractures and pneumocephalus were recorded separately. For report-based extraction, a finding not documented was coded as not reported as present; this did not establish independently verified absence.

The intracranial endpoint was CT-documented traumatic intracranial hemorrhage on the index examination, including epidural hematoma, subdural hematoma, hemorrhagic contusions or cortical hemorrhagic foci, and intraventricular hemorrhage. Multiple hemorrhage types could coexist. Cranial fractures, pneumocephalus, edema, and midline shift did not independently qualify for this endpoint. GCS score bands were reported as neurological severity strata; a GCS score band alone was not used to establish a clinical TBI diagnosis.

### 2.5. Management and Hospital Stay

Initial management was categorized as conservative care, other operative intervention at the study institution, sinus-specific operation at that institution, or direct transfer from the emergency department. Other operative interventions included facial fracture procedures and soft-tissue procedures and were distinguished from a documented sinus-directed procedure. No sinus-specific operation was identified after procedure reconciliation. Transfer was a disposition category; definitive management at the receiving institution was unavailable. Transfer indications included intracranial bleeding, specialist correction of a comminuted sinus fracture, and urgent neurosurgical treatment of a separate comminuted and depressed cranial fracture.

LOS was calculated only for the 30 non-transferred patients. Zeros entered for transferred patients were treated as structurally unavailable LOS, not as zero-day hospital stays. The reported LOS therefore measures stay at the study institution, not total hospitalization after transfer.

### 2.6. Bias

Report-based ascertainment may undercount findings omitted from the original report. Reporting completeness could vary with reader, injury severity, sinus group, age, and CT coverage; uniform ascertainment was not assumed. The 65 exclusions with unknown fracture status could conceal eligible fractures. Reports that addressed intracranial findings only may have concealed sinus fractures in children with intracranial injury, and records without mechanism or GCS score could not be compared with the included ones. Consequently, the direction of any resulting bias is unknown. Complete image rereading and independent duplicate report extraction were not undertaken, so imaging and extraction agreement could not be quantified.

### 2.7. Statistical Analysis

Statistical analysis was performed using SPSS version 31.0.4.0 (IBM Corp., Armonk, NY, USA). Analyses were primarily descriptive, reflecting the study objective of characterizing documented fracture patterns and associated findings in this small retrospective cohort. Categorical variables were summarized as absolute frequencies and percentages, with denominators specified for the complete cohort and each subgroup. Wilson score 95% confidence intervals were calculated for fracture distribution, morphology features, hemosinus, associated imaging findings, residence-specific injury mechanisms, and hemorrhage proportions, and the proportion of patients with posterior table involvement who had traumatic intracranial hemorrhage. The Wilson method was selected because it provides bounded intervals and is suitable for small samples and proportions close to zero or one.

The distribution of continuous variables was assessed using the Shapiro–Wilk test. Median, interquartile range (IQR), and range were used to describe non-normal distributions; mean and sample standard deviation (SD) were additionally reported to characterize dispersion and facilitate comparison with published studies. Quartiles were calculated using linear interpolation. Length of stay was analyzed only for non-transferred patients, because subsequent hospitalization at receiving institutions was unavailable; zero entries for transferred patients were therefore treated as structurally unavailable observations rather than zero-day stays.

All eligible available cases were included without an a priori sample size or power calculation. Subgroup comparisons were descriptive; no hypothesis tests of between-group differences, adjusted association analyses, or predictive models were performed. This approach reflected the small subgroup sizes and the descriptive study objectives. Missing or unknown values were not imputed.

## 3. Results

### 3.1. Participants

**Figure 2:**
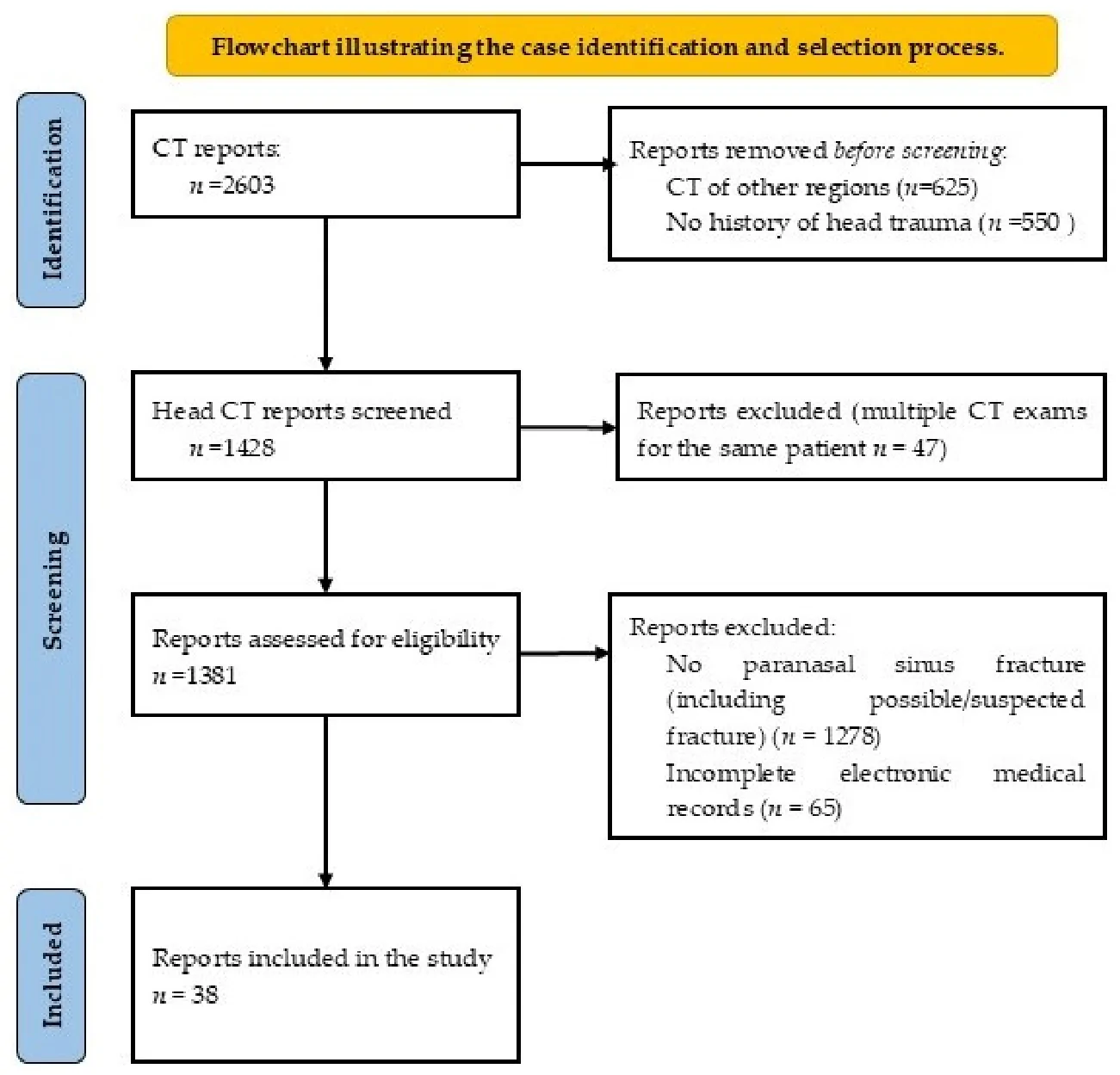
Patient selection flowchart. Of the 2603 head CT examinations performed during the study period, 1428 were performed in a trauma setting. Of these, 1390 were excluded for multiple exams for the same patient (*n* = 47), no paranasal sinus fracture was reported, including possible or suspected fractures (*n* = 1278), and records were excluded as incomplete (missing mechanism of injury or GCS score, or CT report without mention of fractures; *n* = 65), respectively, yielding a final cohort of 38 patients.

The screening process yielded 38 patient records with documented paranasal sinus fractures. Figure 2 presents the detailed patient cohort flowchart.

### 3.2. Cohort Characteristics

A total of 38 pediatric patients (aged 3–17 years) with paranasal sinus fractures documented on CT reports were identified over the five-year study period (2021–2025). The cohort was predominantly male (*n* = 25; 65.8%), with a median age of 13 years (IQR 9.25–15 years; mean 12.0 ± 4.36 years). Age distribution was non-normal (Shapiro–Wilk W = 0.894, *p* = 0.002), with negative skewness reflecting a predominance of older school-age children and adolescents; 13 patients (34.2%) were aged 15 years or older. Rural residence was recorded in 21 patients (55.3%).

Annual case accrual ranged from 4 to 11 cases, with the highest volume recorded in 2025 (*n* = 11; 28.9%) and the lowest in 2021 (*n* = 4; 10.5%). Seasonal distribution demonstrated a winter nadir (*n* = 5; 13.2%), with comparable frequencies across spring (*n* = 12; 31.6%), summer (*n* = 11; 28.9%), and autumn (*n* = 10; 26.3%). Cohort demographic and clinical characteristics are summarised in Table 1.

### 3.3. Fracture Distribution and Extent

Maxillary fractures were documented in 31 patients (81.6%), frontal fractures in 13 (34.2%), ethmoid fractures in 12 (31.6%), and sphenoid fractures in four (10.5%). Posterior table fractures occurred in seven patients (18.4% of the cohort; 53.8% of frontal fracture cases). Right-only and left-only maxillary involvement occurred in 15 and 13 patients, respectively, with bilateral involvement in three. Six of 12 ethmoid fracture cases were bilateral. All four sphenoid fracture cases were left-sided. Detailed fracture and imaging-finding laterality is presented in Table 2; morphology and other associated findings are summarized in Table 3.

Eighteen patients (47.4%) had isolated maxillary involvement, three (7.9%) had isolated frontal involvement, and 17 (44.7%) had multisinus fractures. Twelve patients had two fractured sinus groups, and five had three; no patient had four-group fractures. Bilateral injury did not increase the number of groups. Table 4 presents demographic, clinical, and imaging findings for isolated maxillary, isolated frontal, two-group, and three-group fractures, and Table 5 describes multisinus combinations.

### 3.4. Mechanisms and Residence

Motor vehicle accidents accounted for 19 cases (50.0%): 10 passengers and nine pedestrians. Six patients sustained micromobility injuries; all were aged 13–17 years. Falls accounted for six injuries, assault accounted for three, and sports- and animal-related trauma for two each. The two animal-related injuries were equine bites in rural residents. These mechanisms were described without exposure denominators or age-adjusted comparisons. Residence-specific distributions are presented in Table 6.

### 3.5. Morphology and Associated Imaging Findings

Linear features were coded in 31 patients (81.6%), comminution in 17 (44.7%), and depression in 11 (28.9%). These overlapping variables should not be summed as mutually exclusive fracture categories. Maxillary hemosinus was documented in 32 patients (84.2%), frontal hemosinus in nine (23.7%), ethmoid hemosinus in 14 (36.8%), and sphenoid hemosinus in six (15.8%). Hemosinus and fracture involvement were not interchangeable. At the patient level, three patients had maxillary hemosinus without a coded maxillary fracture, and two had maxillary fractures without documented maxillary hemosinus; side-specific concordance was not assumed from these counts. Nasal bone fractures occurred in 10 patients, pneumocephalus in nine, and orbital content herniation in four. Table 3 summarizes morphology and the nonlateralized associated findings. Figure 1 shows bilateral sphenoid hemosinus in a patient with a fracture of one sphenoid sinus wall only.

### 3.6. Traumatic Intracranial Hemorrhage

Traumatic intracranial hemorrhage was documented in 10 patients (26.3%; 95% CI 15.0–42.0%). Epidural hematoma occurred in four, subdural hematoma in six, hemorrhagic contusions or cortical hemorrhagic foci in five, and intraventricular hemorrhage in one; five patients had multiple hemorrhage types. Four of 18 isolated maxillary cases and six of 17 multisinus cases had hemorrhage; none of the three isolated frontal cases had this. All five patients with GCS ≤8 had hemorrhage.

Two of seven patients with posterior table involvement had hemorrhage (28.6%; 95% CI 8.2–64.1%). Eight of 31 patients without reported posterior table involvement had hemorrhage (25.8%). Within the 13 frontal fracture cases, hemorrhage occurred in two of seven with posterior table involvement and one of six without it. These small descriptive counts do not establish predictive performance. Eight of the 10 hemorrhage cases lacked posterior table involvement (Table 7).

### 3.7. Initial Management and Hospital Stay

Twenty patients (52.6%) received conservative management, and 10 (26.3%) underwent other operative interventions, including facial fracture procedures and soft-tissue debridement or repair. No sinus-specific operation was documented at the study institution. Eight patients (21.1%) were transferred directly from the emergency department. Six transferred patients had traumatic intracranial hemorrhage. Two additional documented transfer indications were a comminuted sinus fracture requiring specialist surgical correction and a comminuted and depressed cranial fracture requiring urgent neurosurgical intervention without coded hemorrhage. Subsequent surgery and outcomes at the receiving institution were unavailable.

Among 30 non-transferred patients, median LOS was 6.0 days (IQR 3.0–7.75; range 1–22; mean 6.3 ± 4.43 days). Median LOS was 3.5 days among 16 isolated maxillary cases, 9.0 days among two isolated frontal cases, and 6.5 days among 12 multisinus cases. The isolated maxillary group included a 22-day admission with substantial associated cranial and hemorrhagic injury. LOS therefore cannot be attributed to sinus extent alone.

## 4. Discussion

This study adds a sinus-specific description to the pediatric craniofacial trauma literature. Earlier series report facial fractures by bone or craniofacial region [5,6,7]. Other studies confine themselves to the frontal sinus [18,19,20,21]. We describe all four sinus groups in one cohort, counting each patient once per group, so that the groups can be read against a single denominator. Two practical points follow for the radiologist reporting these examinations. A sinus fracture on a head CT requires assessment of the intracranial compartment, regardless of sinus group, and whether the posterior table is involved. A wall fracture should also be reported separately from sinus fluid, because the two did not always coincide. These observations come from a selected, report-based cohort and require confirmation with standardized image review.

### 4.1. Fracture Distribution and Developmental Anatomy

Maxillary involvement predominated, occurring in 31 of 38 patients, followed by frontal involvement in 13 and ethmoid involvement in 12. Four patients had sphenoid fractures. Multisinus involvement occurred in 17 patients, comprising 12 two-group and five three-group injuries. These proportions characterize children selected for documented sinus fractures; they do not measure fracture incidence or the probability of a sinus fracture among all children with head trauma.

The maxillary sinus shares boundaries with the orbit and surrounding midfacial skeleton, and sinus-wall fractures may accompany injuries to adjacent facial bones [27]. Nevertheless, the predominance of maxillary involvement in this cohort cannot establish intrinsic biomechanical vulnerability. In broader pediatric facial trauma studies, fracture sites are generally reported as the mandible, orbit, nasal bones, or maxillary and zygomatic regions [3,6]. Those categories differ from the sinus-group variables used here, limiting direct quantitative comparisons.

Mechanism-specific and surgical cohorts further illustrate the importance of anatomical definitions and selection. Oleck et al. described orbital, nasal, and mandibular fractures in children injured in falls, with orbital and frontal sinus fractures occurring more frequently after falls than after other mechanisms [28]. Gąsiorowski et al. identified orbital floor fractures as the most frequent injury in a pediatric craniofacial cohort, with operative care common in that referral population [29]. These studies support contextual discussion of facial trauma but do not provide equivalent four-group sinus fracture frequencies.

Developmental imaging studies remain relevant to case definition. Scuderi et al. described normal pneumatization appearances that can create interpretive difficulties, while Adibelli et al. characterized age-related development across all sinus groups [9,11]. Sardi et al. demonstrated marked variation in frontal sinus growth and its relationship to surrounding bone, and Rennert et al. documented maturation of the sphenoid and parasellar region [12,13]. These data support anatomical confirmation rather than a rigid age threshold. Documented injuries involving an identifiable frontal sinus wall in younger children or in a hypoplastic sinus were therefore retained. Without independent image review or systematic pneumatization measurements, the present series cannot establish age-specific biomechanical susceptibility.

Age needs a separate comment. The cohort ranged from 3 to 17 years, with a median of 13 years, and 13 patients were 15 years or older, so many of the injuries we describe belong to late childhood and adolescence, when frontal sinus pneumatization is advanced but still variable [11,12]. In younger children, a frontal bone fracture may lie over little or no pneumatized sinus, which is why we required documented wall involvement before coding a frontal sinus fracture. We did not stratify the results by age: the 13 frontal fractures would be spread over strata too small to describe, and without independent image review or measurement of pneumatization, we cannot relate fracture pattern or diagnostic difficulty to the stage of sinus development.

The frequent maxillary–ethmoid combination is anatomically plausible, given the ethmoid contribution to the medial orbital wall and its proximity to adjacent facial structures [30]. However, anatomical continuity alone does not prove a particular force-transmission pathway. Bilateral fractures were counted as involvement of one sinus group, preserving the distinction between laterality and anatomical extent. No four-group fractures were recorded; the small numbers in each anatomical stratum preclude inference of a progression in injury severity with increasing sinus extent.

Sphenoid involvement warrants attention because of its skull base location, but the four left-sided cases here are insufficient for a mechanism or laterality inference. Cantini Ardila et al. described sphenoid bone and sinus fractures in an adult facial trauma population, often in association with other facial injuries and higher-energy trauma [31]. Their combined bone-and-sinus endpoint and adult population differ from the present pediatric sinus-wall definition. A separate CT anatomical study by Dahal et al. described carotid canal relationships and sphenoid variants relevant to operative planning; it does not establish vascular injury risk after pediatric trauma [32]. Mao et al. evaluated traumatic carotid-cavernous fistula and emphasized venous engorgement and lateral impact as diagnostic predictors [33]. These reports identify distinct anatomical and vascular considerations, but vascular injury was not systematically assessed in this series.

### 4.2. Posterior Table Involvement and Traumatic Intracranial Hemorrhage

Posterior table fractures were documented in seven patients, two of whom had traumatic intracranial hemorrhage. The hemorrhage proportion was 28.6% with posterior table involvement and 25.8% without reported involvement. Within the 13 frontal fracture cases, hemorrhage occurred in two of seven patients with posterior table injury and one of six without it. These small descriptive comparisons do not establish predictive performance. The principal observation is that eight of the 10 hemorrhage cases had no reported posterior table fracture; posterior table integrity cannot substitute for assessment of the intracranial compartment.

Some complications of frontal sinus fractures emerge after discharge, making follow-up relevant after both conservative and surgical management [34]. Hsieh et al. name contour deformity, sinus dysfunction, cerebrospinal fluid leak, chronic sinusitis, and mucocele formation as the long-term problems that frontal sinus fracture management aims to prevent [35]. In children, the multicenter series of Sobrero et al., which included patients aged 16 years or younger who were operated on for frontal fractures, with at least six months of follow-up, found residual contour deformity of the frontal convexity to be the most frequent complication [36]. Because this series included only children who underwent an operation, it informs surgical outcomes but does not establish the comparative effectiveness of observation and operative treatment [36]. The intracranial sequelae are reported mainly as single cases: Kubilay et al. described delayed pneumocephalus with meningoencephalocele in a 13-year-old boy after facial trauma with defects of the posterior frontal sinus wall, sphenoid, and anterior ethmoid [37], and Jesus et al. described cerebrospinal fluid rhinorrhea that began four years after craniofacial trauma in a 13-year-old girl, with an ethmoid roof encephalocele and the attendant risk of meningitis [38]. Pediatric frontoethmoidal mucoceles are rare and present with subtle signs, and only one of the six children in a recent series had a history of trauma [39], so we cannot say how often a mucocele follows a pediatric frontal fracture. In our cohort, seven children had posterior table involvement; we did not collect long-term outcomes, and eight transferred children left our records. We therefore consider that posterior table involvement, whether or not hemorrhage was documented, justifies follow-up beyond the index admission, and its yield in children needs a prospective design with predefined clinical and imaging follow-up.

Published pediatric frontal series describe injuries with differing neurological and anatomical definitions. Whatley et al. reported substantial associated intracranial injury and cerebrospinal fluid leakage in a small frontal sinus cohort, while Wright et al. described associated head and neck fractures and central nervous system injury in patients aged 6–19 years [19,21]. Vu et al. linked markedly displaced table fractures to nasofrontal outflow tract involvement, cerebrospinal fluid leak, and other indicators of injury severity [18]. Lopez et al. distinguished frontal bone, orbital roof, and sinus-related patterns when developing a pediatric treatment algorithm [20]. These findings support detailed anatomical assessment, but their broader injury endpoints should not be treated as rates of CT-documented hemorrhage.

The adult frontal literature similarly addresses outcomes that are not interchangeable. Pope et al. examined clinical TBI in patients with frontal sinus fractures [17]. Stanwix et al. emphasized CT criteria for nasofrontal outflow tract injury and obstruction [40]. Choi et al. described frequent concurrent central nervous system injury in posterior table fractures and outcomes of selected conservative management [41]. More recent studies support selective management based on fracture displacement, outflow tract status, and dural injury. De Guzman et al. reported conservative treatment in 149 of 201 patients, while displaced fractures, outflow tract injury, cerebrospinal fluid leakage, and adjacent pneumocephalus were more frequent among patients who underwent an operation [42]. Dennis et al. documented frequent sinus reaeration following observation of nonobstructive outflow tract injuries [43]. A recent systematic review also evaluated observation, endoscopic repair, and other noncranialization strategies for displaced posterior table fractures [44]. These findings inform treatment selection but do not establish comparative effectiveness in children. Long-term studies of predominantly adult populations also identify cosmetic satisfaction, forehead sensation, and surgical scarring as relevant outcomes when selecting treatment for anterior table fractures [45,46]. Outflow tract obstruction is not intracranial pathology, and clinical TBI or central nervous system injury need not require a positive hemorrhage finding. Neither outflow tract injury nor cerebrospinal fluid leakage was systematically characterized in the present cohort, limiting comparison with these management studies.

Endpoint consistency is especially important in retrospective extraction. Xun et al. documented underreporting of TBI in pediatric craniomaxillofacial trauma, illustrating that clinical recognition and chart documentation can differ [47]. Our endpoint is restricted to hemorrhage explicitly described on CT, which avoids combining surgical urgency, clinical TBI, pneumocephalus, and depressed cranial fractures into one outcome. This restriction also means that CT-negative or nonhemorrhagic TBI cannot be counted. CT and MRI reviews describe complementary intracranial lesion assessment, but no inference regarding MRI-positive injury is possible here [23,25].

A patient may require neurosurgical intervention without meeting the hemorrhage endpoint, as illustrated by the transferred child with a comminuted and depressed cranial fracture. Conversely, hemorrhage may accompany an isolated sinus-group fracture when additional cranial injuries are present. Tavor et al. examined impact location in a selected pediatric cohort requiring neurosurgery after minor-mechanism trauma [48]. Their study reinforces the need to specify the clinical population and intervention endpoint; it does not establish the causal route of hemorrhage in our patients. The associations between midface injury, neurological status, and hemorrhage reported by Hoppe et al. provide broader clinical context, but our sample is too small for an adjusted analysis [4].

### 4.3. Mechanisms and Demographic Context

Motor vehicle accidents accounted for half of the cohort, with passenger and pedestrian injuries both represented. Imahara et al. identified motor vehicle collisions as the leading mechanism in a national pediatric facial fracture survey, and Patel et al. reported age-dependent mechanisms and fracture patterns in a more recent national analysis [3,7]. The present findings are compatible with the contribution of road traffic trauma to craniofacial injury, but differences in inclusion criteria and anatomical endpoints prevent direct equivalence. Restraint use was not collected, so external evidence on restraint protection cannot be used to explain the individual injuries in this series.

The adolescent predominance and male majority are consistent with demographic patterns described in broader pediatric craniofacial research [3,7]. Irgebay et al. reported age-dependent differences in mechanisms, fracture distribution, hospitalization, and operative care, including more skull and orbital injuries in younger children and more nasal and zygomaticomaxillary injuries in older children [49]. Starck et al. and Kirvelä et al. also demonstrate variation by setting and time period [6,8]. Such evidence supports age-aware interpretation without establishing an age-specific risk in our selected fracture-positive sample.

Falls caused six injuries in this cohort. The proportion is lower than in a recent pediatric TBI cohort restricted to children aged 12 years or younger, in which falls predominated [2]. Fall-specific facial fracture research also highlights concomitant injury and differences from other mechanisms [28]. The contrasting distributions are expected to depend on age limits, injury definitions, and referral pathways. They do not demonstrate a geographic or institutional cause of the difference.

Micromobility injuries occurred in six patients, all aged 13–17 years. Popescu et al. previously described pediatric epidural hematomas following electric scooter falls at the same institution, providing local context for potentially severe neurological injury [50]. Those case reports concern a different endpoint and should not be assumed to represent independent patients from the present cohort. Hirsch et al. examined pediatric recreational conveyances in national surveillance data and found differences in craniofacial injury according to device type and motorization [51]. Our combined scooter-and-bicycle category cannot reproduce those device-specific comparisons, and helmet use was not recorded.

The two equine bite injuries, both in rural residents, contribute a distinctive mechanism to this small series. Published equine craniofacial studies document associated injuries and variable treatment requirements [52]. German and Irish maxillofacial cohorts emphasize substantial injury among unmounted individuals, commonly after kicks [53,54]. Van Balen et al. showed that injury distribution and apparent severity depend on whether patients are mounted and on the selected clinical population, while Diab and Moore described animal-related facial fractures across pediatric and adult groups [55,56]. These studies contextualize animal trauma but predominantly involve mechanisms other than bites and cannot be used to infer occupational exposure in our children.

Rural residence was recorded in 21 patients and urban residence in 17. Residence-specific mechanism counts describe the included cases, not population risk. The dataset contains no denominators for road exposure, micromobility use, or animal contact and no systematic information on restraint use, helmet use, socioeconomic circumstances, or road conditions. Preventive measures supported by external research may be relevant generally, but their effectiveness and the reasons for the residence distribution were not tested in this study.

### 4.4. Hemosinus and Orbital Findings

Hemosinus was common but did not correspond exactly to coded fractures. At the patient level, three children had maxillary hemosinus without a documented maxillary fracture, and two had maxillary fractures without documented maxillary hemosinus. This discordance requires separate coding of fluid and osseous injury, and it cannot identify whether report omission, adjacent hemorrhage, mucosal injury, or other factors explain the finding. Bilateral fluid also does not establish bilateral fractures.

Lambert et al. described the clear sinus sign, and Koedwut et al. evaluated its use on head CT for excluding paranasal sinus fractures [57,58]. Grechushkin et al. found that sinus opacification was more informative for complex facial fractures than for all facial fractures considered together [59]. These diagnostic studies used image assessment and comparison populations that differ from our report-based, fracture-positive cohort. The present data cannot estimate sensitivity, specificity, or predictive values, and apparent fluid–fracture discordance does not validate or refute the sign.

**Figure 3:**
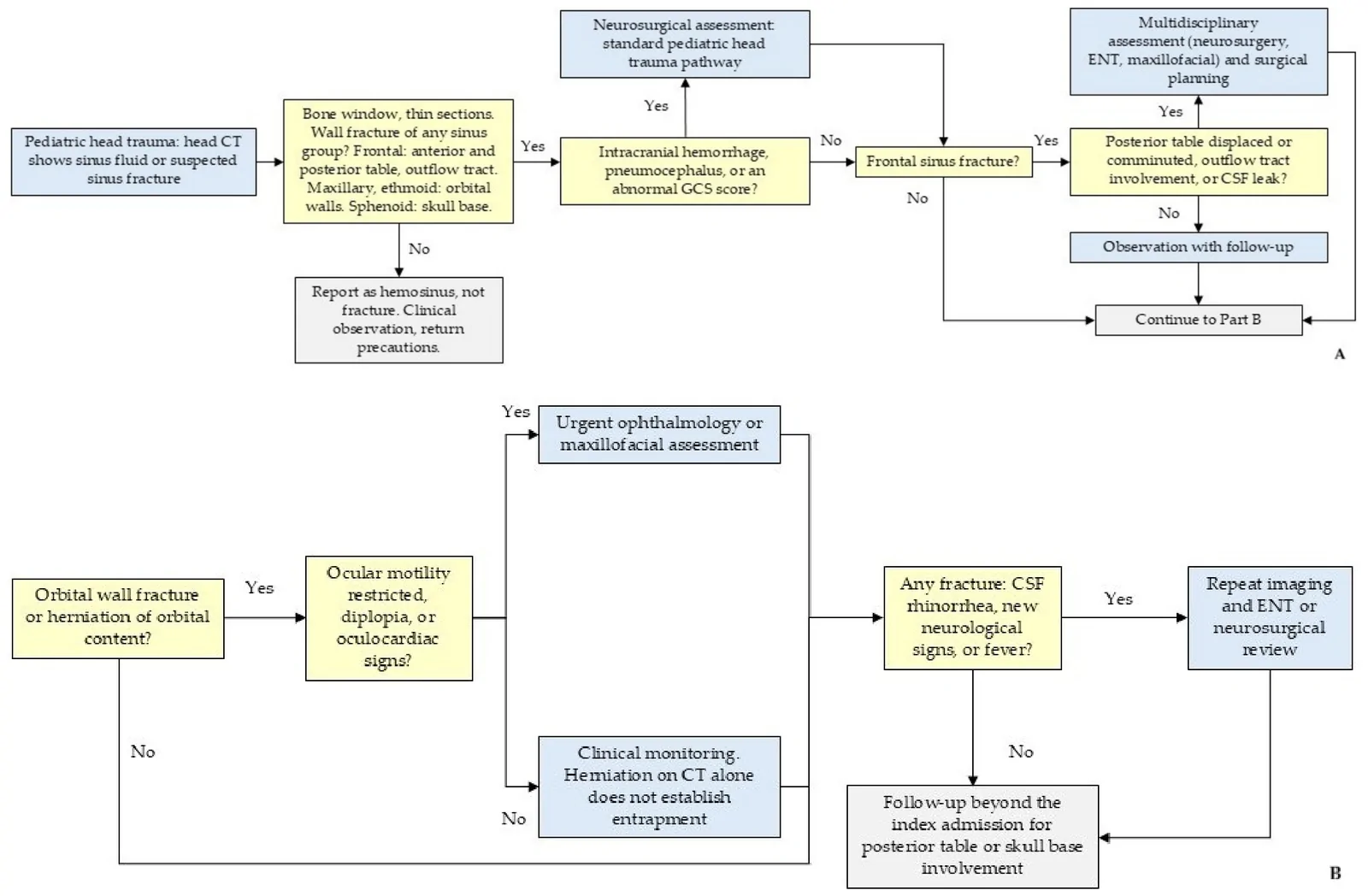
Proposed approach to sinus fluid and suspected sinus fracture on pediatric head CT, combining imaging findings with neurological and ocular status. The pathway is based on published algorithms [14,15,18,20,35,37,38,40,61,62] and was not derived from or validated in the present cohort. CSF, cerebrospinal fluid; CT, computed tomography; ENT, ear, nose, and throat; GCS, Glasgow Coma Scale. (**A**) Imaging triage and frontal sinus pathway. (**B**) Orbital pathway and follow-up. (Yellow-question boxes; blue-action boxes; grey-end boxes).

Huang et al. evaluated head CT findings associated with concomitant orbital floor fractures, including maxillary hemosinus [60]. Pediatric work comparing head and facial CT likewise shows why coverage and clinical examination matter when determining the need for additional facial assessment [24]. These studies support integrating sinus fluid with direct osseous and orbital findings. They do not justify treating hemosinus alone as a fracture diagnosis or assuming that the present extraction captured every orbital floor injury. Hence, a report should state whether a fracture line or wall displacement is demonstrated, and should describe the fluid as hemosinus even when there is no fracture. Figure 3 places this step in a pathway that combines CT findings with neurological and ocular status. The pathway is built from previously published pediatric frontal sinus and orbital fracture algorithms [14,15,18,20,40,61,62].

Orbital content herniation was documented in four patients and was kept distinct from bony displacement and clinical entrapment. Glassman et al. found that CT descriptions of fat or extraocular muscle herniation were poorly predictive of clinical entrapment in children [61]. Guyot et al. reviewed age-related orbital fracture patterns and the particular implications of pediatric trapdoor injury [62]. Koltai et al. similarly described the shift from orbital roof fractures in younger children toward lower orbital fractures in older children [22]. The present database did not systematically record ocular motility or operative confirmation and therefore supports a description of herniation only.

Recent pediatric orbital research has explored modified CT-based indices incorporating fracture type to support assessment of surgical need [63]. No equivalent quantitative assessment was available here. An additional interpretive consideration is nontraumatic lamina papyracea dehiscence: Deniz and Tekinhatun described CT-visible defects, sometimes with herniated orbital tissue, in an adult nontrauma population [64]. Their work concerns a potential fracture mimic rather than traumatic force transmission. It reinforces the value of image-based differentiation in future studies, without establishing that any herniation in our cohort was nontraumatic.

### 4.5. Initial Management and Hospitalization

Twenty patients received conservative management, 10 underwent other operative interventions, and eight were transferred directly from the emergency department. No sinus-specific operation was documented locally. These categories describe initial management and institutional disposition rather than the complete treatment course. In particular, one transfer was for specialist correction of a comminuted sinus fracture, and post-transfer procedures and outcomes were unavailable. The absence of local sinus surgery cannot establish that no patient required sinus-directed treatment.

Pediatric frontal management studies commonly individualize care according to displacement, associated neurological injury, outflow tract involvement, and cerebrospinal fluid leakage [18,20]. Earlier pediatric cohorts likewise describe multidisciplinary management when orbital, dural, or other craniofacial injuries coexist [19,21]. General pediatric facial fracture guidance also considers growth and developing anatomy [10]. However, conservative management in our cohort cannot be attributed to minimal displacement or judged effective because displacement, treatment indications, and long-term outcomes were not collected consistently.

The broader frontal literature describes both sinus-preserving management and operative options, with decisions based on separate anatomical subsites and the associated injury burden [14,15]. Vasconcelos et al. provide an adult multidisciplinary series in which conservative and operative strategies were selected according to fracture characteristics, and Choi et al. describe conservative treatment of selected posterior table injuries [16,46]. These studies supply treatment context but do not validate pediatric management decisions or the safety of local conservative care in this dataset.

Hospital stay was observable only for the 30 non-transferred patients. Median LOS was 3.5 days for isolated maxillary fractures, 9.0 days for isolated frontal fractures, and 6.5 days for multisinus fractures. The isolated frontal estimate is based on two patients, while the isolated maxillary subgroup includes a 22-day admission with substantial cranial and hemorrhagic injury. These values do not establish a monotonic relationship between sinus extent and hospitalization, and transfer removes patients with potentially different injury severity from local LOS observation.

Lopez et al. analyzed hospital stay in relation to defined frontal fracture patterns, while Perez-Otero et al. examined acute hospital course across broader craniofacial categories in a large national dataset [5,20]. Their analyses concern injury definitions and severity covariates not reproduced here. Schaller et al. documented frequent associated injuries and polytrauma in children with anterior skull base fractures, reinforcing why sinus extent alone is an incomplete representation of the injury burden [65]. Direct numerical comparison of LOS across these selected populations would be misleading.

Cai et al. explored the relationship between frontal sinus volume and intracranial injury severity in support of the crumple-zone hypothesis [66]. Sinus volume and overall injury severity were not quantified here, so neither an energy-absorption effect nor its relationship to LOS can be evaluated. Future studies should combine standardized image review, sinus development and wall-specific measurements, overall injury severity, definitive post-transfer care, and complete hospitalization and functional follow-up. This would permit analyses that distinguish anatomical extent from the neurological and systemic injuries determining clinical outcomes.

## 5. Limitations

This single-center retrospective series is small and selected through existing radiology reports. Independent image review was unavailable, and CT acquisition parameters, coverage, and reconstruction details were not systematically captured in the analytic database. Findings not documented in a report may have been missed, with unknown differential effects across ages, readers, mechanisms, and sinus groups. Findings described as possible or suspected were coded as not reported, so the series is limited to fractures documented as definite, and fractures, hemosinus, and associated findings, including hemorrhage, are probably undercounted. Laterality coding was reconciled against the clinical information on the CT request form before analysis, but independent extraction reliability and image interpretation agreement were not assessed. The cohort comes from one pediatric emergency hospital in Romania and reflects local referral and transfer pathways, trauma care, CT protocols, and reporting habits, any of which may differ elsewhere. The records were created for clinical care, not for research, so the reports’ findings determined what could be coded. The 38 children are a selected fracture-positive group, and their proportions should not be generalized to other institutions or to children with head trauma as a whole.

The 65 excluded records had unknown fracture status. Their exclusion may have omitted eligible injuries, and no claim of complete fracture ascertainment is justified. Screening counts describe examinations rather than a population at risk. Frontal pneumatization, wall-specific displacement, outflow tract injury, cerebrospinal fluid leak, ocular entrapment, and overall injury severity were not systematically quantified. Morphology was recorded at the patient level across sinus and contiguous facial fractures and cannot be assigned to every individual sinus.

Residence and mechanism comparisons are unadjusted descriptions with small cells and no exposure denominators. LOS excludes transferred patients and does not represent total hospitalization. Definitive management and long-term functional or sinus-related outcomes after transfer were unavailable. The study cannot establish incidence, diagnostic accuracy, causal mechanisms, independent predictors, or the safety of conservative management.

## 6. Conclusions

In this five-year report-based series of 38 children with documented paranasal sinus fractures, maxillary involvement was most frequent and 17 patients had multisinus fractures. Traumatic intracranial hemorrhage was documented in 10 patients and occurred in both isolated maxillary and multisinus cases; eight hemorrhage cases lacked reported posterior table fractures. Initial management included conservative care, other operative interventions, and transfer, with no sinus-specific operation documented locally. These findings provide a descriptive account of the selected cohort and warrant validation using standardized image review, explicit anatomical definitions, and complete treatment and follow-up data.

## Figures and Tables

**Table 1:** Cohort demographic and clinical characteristics (*n* = 38).

Variable	*n*	% (*n* = 38)
Year of presentation		
2021	4	10.5
2022	8	21.1
2023	10	26.3
2024	5	13.2
2025	11	28.9
Season		
Spring	12	31.6
Summer	11	28.9
Autumn	10	26.3
Winter	5	13.2
Sex		
Male	25	65.8
Female	13	34.2
Residence		
Rural	21	55.3
Urban	17	44.7
Mechanism of injury		
MVA—passenger	10	26.3
MVA—pedestrian	9	23.7
Micromobility	6	15.8
Fall from height	4	10.5
Assault	3	7.9
Fall from standing height	2	5.3
Sports	2	5.3
Animal-related	2	5.3
GCS at admission		
13–15	31	81.6
9–12	2	5.3
≤8	5	13.2
Management		
Conservative	20	52.6
Operative—not sinus-related	10	26.3
Transfer to another center	8	21.1
Operative—sinus-related procedure	0	0.0

GCS, Glasgow Coma Scale; TBI, traumatic brain injury; MVA, motor vehicle accident.

**Table 2:** Laterality of paranasal sinus fracture and hemosinus (*n* = 38).

Sinus/Variable	Any *n* (%)	95% CI	Right *n* (%)	Left *n* (%)	Bilateral *n* (%)
**Fracture Distribution**					
Maxillary sinus	31 (81.6)	66.6–90.8	15 (39.5)	13 (34.2)	3 (7.9)
Frontal sinus	13 (34.2)	21.2–50.1	4 (10.5)	5 (13.2)	4 (10.5)
Posterior table involvement	7 (18.4)	9.2–33.4	2 (5.3)	3 (7.9)	2 (5.3)
Ethmoid	12 (31.6)	19.1–47.5	2 (5.3)	4 (10.5)	6 (15.8)
Sphenoid	4 (10.5)	4.2–24.1	0 (0.0)	4 (10.5)	0 (0.0)
**Hemosinus**					
Maxillary	32 (84.2)	69.6–92.6	13 (34.2)	14 (36.8)	5 (13.2)
Ethmoid	14 (36.8)	23.4–52.7	3 (7.9)	4 (10.5)	7 (18.4)
Frontal	9 (23.7)	13.0–39.2	4 (10.5)	3 (7.9)	2 (5.3)
Sphenoid	6 (15.8)	7.4–30.4	0 (0.0)	4 (10.5)	2 (5.3)

95% CI, confidence interval (Wilson score method). Right, left, and bilateral values are mutually exclusive.

**Table 3:** Fracture morphology and other associated imaging findings.

Finding	Any *n* (%)	95% CI
**Fracture morphology**		
Linear	31 (81.6)	66.6–90.8
Comminuted	17 (44.7)	30.1–60.3
Depressed	11 (28.9)	17.0–44.8
**Associated imaging findings**		
Nasal bone fracture	10 (26.3)	15.0–42.0
Traumatic intracranial hemorrhage	10 (26.3)	15.0–42.0
Pneumocephalus	9 (23.7)	13.0–39.2
Orbital content herniation	4 (10.5)	4.2–24.1

*n* = 38. Morphology features overlap and describe sinus and contiguous facial skeletal fractures at the patient level; morphology attributable solely to separate neurocranial fractures is excluded. These are not mutually exclusive categories. CI, confidence interval; Wilson score method.

**Table 4:** Demographic, clinical, and imaging characteristics by sinus fracture extent.

Variable	Isolated Maxillary *n* = 18 (%)	Isolated Frontal *n* = 3 (%)	2-Sinus Groups *n* = 12 (%)	3-Sinus Groups *n* = 5 (%)
Sex				
Male	12 (66.7)	2 (66.7)	8 (66.7)	3 (60.0)
Female	6 (33.3)	1 (33.3)	4 (33.3)	2 (40.0)
Residence				
Rural	11 (61.1)	1 (33.3)	7 (58.3)	2 (40.0)
Urban	7 (38.9)	2 (66.7)	5 (41.7)	3 (60.0)
Mechanism of injury				
MVA—pedestrian	4 (22.2)	1 (33.3)	3 (25.0)	1 (20.0)
Micromobility	3 (16.7)	0 (0.0)	1 (8.3)	2 (40.0)
Assault	3 (16.7)	0 (0.0)	0 (0.0)	0 (0.0)
MVA—passenger	2 (11.1)	1 (33.3)	6 (50.0)	1 (20.0)
Fall from standing height	2 (11.1)	0 (0.0)	0 (0.0)	0 (0.0)
Sports	2 (11.1)	0 (0.0)	0 (0.0)	0 (0.0)
Fall from height	1 (5.6)	0 (0.0)	2 (16.7)	1 (20.0)
Animal-related	1 (5.6)	1 (33.3)	0 (0.0)	0 (0.0)
GCS at admission				
13–15	16 (88.9)	3 (100.0)	9 (75.0)	3 (60.0)
9–12	1 (5.6)	0 (0.0)	0 (0.0)	1 (20.0)
≤8	1 (5.6)	0 (0.0)	3 (25.0)	1 (20.0)
Fracture morphology				
Linear	14 (77.8)	2 (66.7)	10 (83.3)	5 (100.0)
Comminuted	8 (44.4)	1 (33.3)	6 (50.0)	2 (40.0)
Comminuted + depressed	7 (38.9)	0 (0.0)	3 (25.0)	1 (20.0)
Posterior table involvement				
Present	0 (0.0)	1 (33.3)	4 (33.3)	2 (40.0)
Absent/not reported	18 (100.0)	2 (66.7)	8 (66.7)	3 (60.0)
Traumatic intracranial hemorrhage				
Present	4 (22.2)	0 (0.0)	4 (33.3)	2 (40.0)
Absent/not reported	14 (77.8)	3 (100.0)	8 (66.7)	3 (60.0)
Management				
Conservative	10 (55.6)	1 (33.3)	6 (50.0)	3 (60.0)
Operative—sinus-related	0 (0.0)	0 (0.0)	0 (0.0)	0 (0.0)
Operative—not sinus-related	6 (33.3)	1 (33.3)	3 (25.0)	0 (0.0)
Transfer to another center	2 (11.1)	1 (33.3)	3 (25.0)	2 (40.0)
LOS, days				
LOS available n	16	2	9	3
Median (IQR)	3.5 (2.75–6.00)	9.0 (7.50–10.50)	6.0 (6.00–9.00)	8.0 (5.50–9.50)
Mean ± SD	5.25 ± 5.16	9.00 ± 4.24	7.11 ± 3.10	7.33 ± 4.04
Range	1–22	6–12	3–13	3–11

Values are *n* (%) unless otherwise specified; percentages use the column-specific denominator. “Isolated” refers only to sinus groups; other injuries may coexist. Morphology features overlap. Findings not documented were not coded as present. LOS excludes transferred patients; IQR uses the 25th and 75th percentiles, and SD is the sample standard deviation. GCS, Glasgow Coma Scale; MVA, motor vehicle accident; LOS, length of stay; IQR, interquartile range; SD, standard deviation.

**Table 5:** Multisinus fracture combinations.

Sinus Groups	Number of Groups	*n*
Maxillary + Ethmoid	2	5
Frontal + Ethmoid + Sphenoid	3	2
Frontal + Ethmoid	2	2
Maxillary + Sphenoid	2	2
Maxillary + Frontal + Ethmoid	3	3
Maxillary + Frontal	2	3

Two-group cases: *n* = 12; three-group cases: *n* = 5. LOS was available for nine and three patients, respectively; medians were 6.0 days (range 3–13) and 8.0 days (range 3–11). No four-group fractures were recorded.

**Table 6:** Injury mechanism and traumatic intracranial hemorrhage by residence (*n* = 38).

Mechanism	Total *n*	Urban *n* (%)	Urban 95% CI	Rural *n* (%)	Rural 95% CI
MVA—passenger	10	2 (11.8)	3.3–34.3	8 (38.1)	20.8–59.1
MVA—pedestrian	9	4 (23.5)	9.6–47.3	5 (23.8)	10.6–45.1
Micromobility	6	4 (23.5)	9.6–47.3	2 (9.5)	2.7–28.9
Fall from height	4	3 (17.6)	6.2–41.0	1 (4.8)	0.8–22.7
Assault	3	2 (11.8)	3.3–34.3	1 (4.8)	0.8–22.7
Animal-related	2	0 (0.0)	0.0–18.4	2 (9.5)	2.7–28.9
Sports	2	2 (11.8)	3.3–34.3	0 (0.0)	0.0–15.5
Fall from standing height	2	0 (0.0)	0.0–18.4	2 (9.5)	2.7–28.9
Traumatic intracranial hemorrhage	10	4 (23.5)	9.6–47.3	6 (28.6)	13.8–50.0

Residence percentages and Wilson score 95% confidence intervals use the respective urban (*n* = 17) and rural (*n* = 21) denominators and describe the selected cohort, not population risk. CI, confidence interval.

**Table 7:** Traumatic intracranial hemorrhage by posterior table status and admission GCS.

Stratum	Total N	Hemorrhage *n* (%)	No Hemorrhage *n*
Posterior table present	7	2 (28.6)	5
Posterior table absent/not reported	31	8 (25.8)	23
GCS 13–15	31	3 (9.7)	28
GCS 9–12	2	2 (100.0)	0
GCS ≤ 8	5	5 (100.0)	0

Posterior table and GCS rows are separate stratifications of the same cohort. Counts do not constitute an adjusted association or diagnostic validation.

## Data Availability

The data presented in this study are available on request from the corresponding author. The data are not publicly available due to privacy and ethical restrictions.

## Supplementary Materials

The following supporting information can be downloaded at https://www.mdpi.com/article/10.3390/diagnostics16213638/s1. Table S1: STROBE checklist.

## Abbreviations

The following abbreviations are used in this manuscript:

CT	Computed tomography
MRI	Magnetic resonance imaging
TBI	Traumatic brain injury
STROBE	Strengthening the Reporting of Observational Studies in Epidemiology
PACS	Picture archiving and communication system
GCS	Glasgow Coma Scale
LOS	Length of stay
IQR	Interquartile range
SD	Standard deviation
MVA	Motor vehicle accident
CI	Confidence interval
