# Supplementary material for: Paranasal Sinus Fractures in Pediatric Head Trauma: A Retrospective CT-Based Analysis of Pattern Distribution, Mechanism Correlation and Associated Intracranial Injury
Source: Diagnostics (Basel). 2026 Nov 4;16(21):3638. doi: 10.3390/diagnostics16213638 (PMC13762297; doi:10.3390/diagnostics16213638)
Supplement: Supplementary file 1 [file diagnostics-16-03638-s001.zip › diagnostics-4653618-supplementary.pdf]

Supplementary Table S1. STROBE checklist

	Item No	Recommendation	Location in manuscript
**Title and abstract**	1	(*a*) Indicate the study's design with a commonly used term in the title or the abstract	Abstract page 1
		(*b*) Provide in the abstract an informative and balanced summary of what was done and what was found	Abstract page 1
**Introduction**			
Background/rationale	2	Explain the scientific background and rationale for the investigation being reported	Introduction page 2
Objectives	3	State specific objectives, including any prespecified hypotheses	Introduction pages 1-2
**Methods**			
Study design	4	Present key elements of study design early in the paper	Section 2.1 page 3
Setting	5	Describe the setting, locations, and relevant dates, including periods of recruitment, exposure, follow-up, and data collection	Section 2.1 page 3
Participants	6	(*a*) Give the eligibility criteria, and the sources and methods of selection of participants	Section 2.2 page 3
Variables	7	Clearly define all outcomes, exposures, predictors, potential confounders, and effect modifiers. Give diagnostic criteria, if applicable	Sections 2.3 page 3, 2.4 page 4, and 2.5. page 4
Data sources/ measurement	8*	For each variable of interest, give sources of data and details of methods of assessment (measurement). Describe comparability of assessment methods if there is more than one group	Sections 2.3 page 3, and 2.4 page 4
Bias	9	Describe any efforts to address potential sources of bias	Sections 2.6 pages 4-5, and 5 page 18
Study size	10	Explain how the study size was arrived at	Section 2.7 page 5 (no sample-size calculation)
Quantitative variables	11	Explain how quantitative variables were handled in the analyses. If applicable, describe which groupings were chosen and why	Section 2.7, page 5; GCS bands in Section 2.4, page 4
Statistical methods	12	(*a*) Describe all statistical methods, including those used to control for confounding	Section 2.7, page 5
		(*b*) Describe any methods used to examine subgroups and interactions	Section 2.7, page 5; Table 4, page 8
		(*c*) Explain how missing data were addressed	Sections 2.2, page 3 (65 exclusions), and 2.7, page 5 (no imputation)
		(*d*) If applicable, describe analytical methods taking account of sampling strategy	Section 2.7, page 5 (all eligible cases included)
		(*e*) Describe any sensitivity analyses	Not applicable; none performed (Section 2.7, page 5)
**Results**			
Participants	13*	(a) Report numbers of individuals at each stage of study—eg numbers potentially	Sections 2.2, page 3, and 3.1, page 5;

		eligible, examined for eligibility, confirmed eligible, included in the study, completing follow-up, and analysed	Figure 1, page 6
		(b) Give reasons for non-participation at each stage	Section 2.2, page 3; Figure 1, page 6
		(c) Consider use of a flow diagram	Figure 1, page 6
Descriptive data	14*	(a) Give characteristics of study participants (eg demographic, clinical, social) and information on exposures and potential confounders	Section 3.2 and Table 1, page 6
		(b) Indicate number of participants with missing data for each variable of interest	Sections 2.2, page 3, and 2.5, page 4 (LOS for 30 patients); Table 5 footnote, page 9
Outcome data	15*	Report numbers of outcome events or summary measures	Sections 3.3 to 3.7, pages 7–11; Tables 2, 3, 7
Main results	16	(*a*) Give unadjusted estimates and, if applicable, confounder-adjusted estimates and their precision (eg, 95% confidence interval). Make clear which confounders were adjusted for and why they were included	Tables 2, 3, 6 and 7 (pages 7, 8, 9, 11); no adjusted estimates (Section 2.7, page 5)
		(*b*) Report category boundaries when continuous variables were categorized	GCS bands: Tables 1, page 6, and Table 7, page 11
		(*c*) If relevant, consider translating estimates of relative risk into absolute risk for a meaningful time period	Not applicable
Other analyses	17	Report other analyses done—eg analyses of subgroups and interactions, and sensitivity analyses	Table 4, page 8; Table 6, page 9; Table 7, page 11 (descriptive only)
**Discussion**			
Key results	18	Summarise key results with reference to study objectives	Section 4, first paragraph, page 11; Section 4.2, page 13; Conclusions, page 18
Limitations	19	Discuss limitations of the study, taking into account sources of potential bias or imprecision. Discuss both direction and magnitude of any potential bias	Section 2.6, pages 4–5; Section 5, page 18
Interpretation	20	Give a cautious overall interpretation of results considering objectives, limitations, multiplicity of analyses, results from similar studies, and other relevant evidence	Section 4, pages 11-17
Generalisability	21	Discuss the generalisability (external validity) of the study results	Section 5, page 18
**Other information**			
Funding	22	Give the source of funding and the role of the funders for the present study and, if applicable, for the original study on which the present article is based	Funding statement page 18
